# Thyrotropin and free thyroxine levels and coronary artery disease: cross-sectional analysis of the Brazilian Longitudinal Study of Adult Health (ELSA-Brasil)

**DOI:** 10.1590/1414-431X20177196

**Published:** 2018-03-15

**Authors:** E.J.F. Peixoto de Miranda, M.S. Bittencourt, H.L. Staniak, R. Sharovsky, A.C. Pereira, M. Foppa, I.S. Santos, P.A. Lotufo, I.M. Benseñor

**Affiliations:** 1 Centro de Pesquisa Clínica, Hospital Universitário, Universidade de São Paulo, São Paulo, SP, Brasil; 2 Laboratório de Genética, Instituto do Coração, Faculdade de Medicina, Universidade de São Paulo, São Paulo, SP, Brasil; 3 Faculdade de Medicina, Universidade Federal do Rio Grande do Sul, Porto Alegre, RS, Brasil

**Keywords:** Thyroid disorders, Thyrotropin levels, Coronary artery disease, Coronary computed tomography angiography, Subclinical atherosclerosis, Cardiovascular disease

## Abstract

Data on the association between subclinical thyroid dysfunction and coronary artery disease (CAD) is scarce. We aimed to analyze the association between thyroid function and CAD using baseline data from the Brazilian Longitudinal Study of Adult Health (ELSA-Brasil). We included subjects with normal thyroid function (0.4-4.0 mIU/L, and normal free thyroxine, FT4, or 0.8 to 1.9 ng/dL), subclinical hypothyroidism (SCHypo; TSH>4.0 mIU/L and normal FT4), and subclinical hyperthyroidism (SCHyper; TSH<0.4 mIU/L and normal FT4) evaluated by coronary computed tomography angiography. We excluded individuals using medications that interfere in thyroid function or with past medical history of cardiovascular disease. Logistic regression models evaluated the presence of CAD, segment involvement score (SIS) >4, and segment severity score (SSS) >4 of coronary arteries as the dependent variables, and quintiles of TSH and FT4 as the independent variables, adjusted for demographical data and cardiovascular risk factors. We included 767 subjects, median age 58 years (IQR=55-63), 378 (49.3%) women, 697 euthyroid (90.9%), 57 (7.4%) with SCHypo, and 13 (1.7%) with SCHyper. No association between TSH and FT4 quintiles and CAD prevalence was noted. Similarly, no association between TSH levels and the extent or severity of CAD, represented by SIS>4 and SSS>4 were seen. Restricting analysis to euthyroid subjects did not alter the results. TSH levels were not significantly associated with the presence, extent, or severity of CAD in a middle-aged healthy population.

## Introduction

Thyroid disorders have been associated to coronary heart disease (1–3). Two recent meta-analyses of several cohort studies, one focused on subclinical hyperthyroidism (SCHyper) and another in subclinical hypothyroidism (SCHypo), showed an association of subclinical thyroid disorders with coronary heart disease (CHD) events and all-cause and cardiovascular mortality. Collet et al. (2) showed that SCHyper increased cardiovascular mortality, all-cause mortality and CHD events risk by 29, 24, and 21%, respectively, in comparison to euthyroid subjects. On the other end of the spectrum, Rodondi et al. (3) found that SCHypo increases risk of the same outcomes in subjects with thyrotropin (TSH) above 10.0 mIU/L. In this subgroup, SCHypo increased respectively 89, 22, and 58% the risk of CHD events and all-cause and cardiovascular mortality.

In the last decades, advances in coronary computed tomography angiography (CCTA) have allowed the non-invasive detection of coronary artery disease (CAD) (4). Several studies showed that CAD is an important predictor of future CHD events and mortality (4–6). However, data on the association between CAD detected by CCTA and subclinical thyroid dysfunction is scarce. Only one large retrospective study that evaluated subjects with 10-year CHD risk ≥10% who underwent CCTA found an association between CAD and SCHypo in comparison with euthyroid subjects (7). Data on SCHyper is even scarcer: only one study (8) with 100 subjects referring chest pain, 6 of them with SCHyper, 4 with SCHypo, and 90 euthyroid, were compared for the presence of CAD using invasive angiography. In that study, higher levels of TSH were associated with the severity of CAD, defined as obstruction above 50% in two or three vessels, but no association was found for SCHyper or low TSH levels. (8). Two large South Korean cross-sectional analyses that evaluated CAD measured by coronary artery calcification (CAC) and FT4 found an inverse relationship among euthyroid subjects (9) and euthyroid men (10).

The Brazilian Longitudinal Study of Adult Health (ELSA-Brasil) is a prospective cohort study with data about thyroid function and subclinical atherosclerosis evaluated by CCTA in a subsample of participants. We aimed to evaluate cross-sectionally the association between TSH levels and CAD using the baseline data of the ELSA-Brasil.

## Material and Methods

This study is a cross-sectional analysis using baseline data from ELSA-Brasil. Briefly, the study included 15,105 civil servants, aged 35–74 years from six institutions from six Brazilian cities. Baseline data occurred from August 2008 to December 2010. The study aims to determine the incidence of cardiovascular diseases and diabetes and their associated risk factors. A detailed description of the ELSA-Brasil study can be found elsewhere (11,12). The protocol was approved at all six centers by the Institutional Review Boards addressing research in human participants according to the Declaration of Helsinki (Hospital Research Ethics Commission approval No. 659/06I). Written informed consent was obtained from all participants. The present analysis was restricted to 982 subjects from the Centro de Pesquisa Clínica, Hospital Universitário de São Paulo, where CCTA protocol was performed as an ancillary study in a subsample at baseline examination.

### Thyroid function

The thyroid-stimulating hormone (TSH) and free thyroxine (FT4) were dosed using a third generation immunoenzymatic assay (Siemens, USA) in serum obtained from venous blood samples after an overnight fast and centrifugation. FT4 levels were only evaluated in participants who presented altered TSH levels. In this study, reference range levels were 0.4-4.0 mIU/L for TSH and 0.8–1.9 ng/dL for FT4, similar to those used elsewhere (13,14).

Participants in ELSA-Brasil were classified into five categories of thyroid function: overt hyperthyroidism (low serum TSH and high levels of FT4, or use of medication to treat hyperthyroidism), subclinical hyperthyroidism (low serum TSH, normal levels of FT4, and no use of thyroid drugs), euthyroidism (normal TSH and no use of thyroid drugs), subclinical hypothyroidism (high TSH levels, normal levels of FT4, and no use of thyroid drugs), and overt hypothyroidism (high TSH and low FT4 levels, or use of levothyroxine to treat hypothyroidism).

Here, participants with overt thyroid disorders were excluded and only euthyroid participants or those with subclinical thyroid dysfunction were included in the analysis. We also excluded participants with previous history of cardiovascular disease (angina, myocardial infarction, coronary artery revascularization surgery, stroke, and congestive heart failure) and all patients using drugs that can interfere with thyroid function, such as amiodarone, carbamazepine, carbidopa, phenytoin, furosemide, haloperidol, heparin, interferon, levodopa, lithium, metoclopramide, propranolol, primidone, rifampicin, and valproic acid (15).

### Coronary computed tomography angiography (CCTA)

Participants underwent a CCTA with a 64-detector scanner (Brilliance 64, Philips Healthcare, Netherlands). In the majority of patients, ECG-gated contrast-enhanced prospective CCTA was performed with a collimation of 64×0.625 mm, gantry rotation time 400 ms, with tube current of 150 mA and tube potential of 120 kV. All patients with a heart rate above 60 bpm received oral beta-blockers before the image acquisition. Iodine contrast (Ultravist 370, Bayer, Germany) injected with a dual head injector (Medrad Inc., USA) followed by 60 mL of saline at the same rate. Automated bolus tracking was used by placing a circular region of interest in the descending aorta and acquisition was triggered when the average attenuation value in the region of interest reached 150 Hounsfield units. The 75% RR interval image was used for image reconstruction and coronary analysis. Images were reconstructed using standard filtered back projection kernels. At the physician’s discretion, cases with heart rate above 60 bpm were scanned using retrospective gating with dose modulation and a peak dose window between 40–80% of the cardiac cycle.

The coronary tree was segmented in a 17 segments model (4) and all arteries of at least 1.5 mm were analyzed and classified by experienced cardiologists as having no obstruction (0%), mild to moderate (below 50%), moderately severe (50 to 70%), or severe (>70%) for each segment. The number of obstructed segments was summarized by the segment involvement score (SIS), which is calculated by the sum of the number of segments with obstruction, ranging from 0 to 17 (16) and the segment severity score (SSS), a number that ranges from 0 to 51, and is scored according to degree of disease: 0 for no obstruction, 1 for non-obstructive coronary artery disease, 2 for 50–70% stenosis, and 3 for ≥70% stenosis (17).

We defined presence of CAD as at least one coronary segment out of 17 with any degree of obstruction demonstrated by CCTA. Outcome data were categorized in presence of CAD (*vs* absence of CAD), SIS>4 *vs* SIS≤4 and SSS>4 *vs* SSS≤4.

### Other variables

Each participant underwent an interview at workplace and during a visit to the research center for clinical exams according to a standard protocol (18). The interview and clinical and laboratory examinations were performed by trained personnel with strict quality control. Questionnaires addressed age (presented as median and interquartile range), self-defined race (Black, Brown, White, Asian or Native), and smoking status (never, former, and current). Blood pressure (BP) was taken using a validated oscillometric device, the Omron HEM 705CPINT (Omron Co, Japan). Three measurements were taken at 1-min intervals. The mean of the two latest BP measurements was considered as the value for high BP definition. Height and weight were measured in light clothes using standardized techniques. Body mass index (BMI) was calculated by dividing weight in kilograms by height in squared meters, and absolute 10-year cardiovascular risk was estimated using the Framingham risk score, calculated according to criteria described elsewhere (19).

Hypertension was defined as the use of medications to treat hypertension, a systolic BP≥140 mmHg or a diastolic blood pressure ≥90 mmHg at ELSA-Brasil baseline assessment. Diabetes was defined as a medical history of diabetes mellitus, the use of medications to treat diabetes mellitus, a fasting serum glucose ≥126 mg/dL, HbA1c levels ≥6.5% or a 2-h oral glucose tolerance test ≥200 mg/dL. Dyslipidemia was defined as the use of lipid-lowering treatment or a low-density lipoprotein (LDL) cholesterol level ≥130 mg/dL. Glomerular filtration rate (GFR) was calculated by the equation from the Chronic Kidney Disease Epidemiology Collaboration (CKD-Epi) published elsewhere (20).

### Laboratory tests

Glucose levels were measured using the hexokinase method. The enzymatic colorimetric assay was used to measure total and HDL-cholesterol and triglycerides. LDL-cholesterol was calculated using the Friedewald equation, except for cases with elevated triglyceride levels (>400 mg/dL) when an enzymatic colorimetric assay was used (ADVIA 1200, Siemens). Creatinine was measured using Jaffe's method (ADVIA 1200, Siemens) (21).

### Statistical analysis

Continuous variables are reported as mean and standard deviation or median and interquartile range (IQR) and compared using P for trend obtained by ANOVA (linear polynomial contrast) or Jonckheere-Terpstra trend test as appropriate after assessing of normality assumptions. Categorical variables are reported as proportions and compared using P for trend obtained by linear-by-linear association chi-square test as appropriate.

Logistic regression models were built using presence of CAD (*vs* absence of CAD), SIS>4 (*vs* SIS≤4), and SSS>4 (*vs* SSS≤4) as the dependent variables. TSH and FT4 quintiles were included as independent variables, using the third quintile as reference. This choice is supported by previous studies suggesting a possible association of thyroid disorders and cardiovascular disease considering both extremes of TSH (2,3). Model 1 also included age, sex, and race/skin color. Model 2 included all variables of model 1 plus smoking, diabetes mellitus, dyslipidemia, and hypertension as factors, and BMI, HDL-cholesterol, triglycerides, glomerular filtration rate by CKD-Epi, and high sensitivity C-reactive protein (hs-CRP) as continuous variables. We performed additional analyses restricted to euthyroid subjects. Analyses were done using SPSS 20.0 (IBM, USA). P<0.05 was considered significant and all tests were two-tailed.

## Results

**Figure 1.**
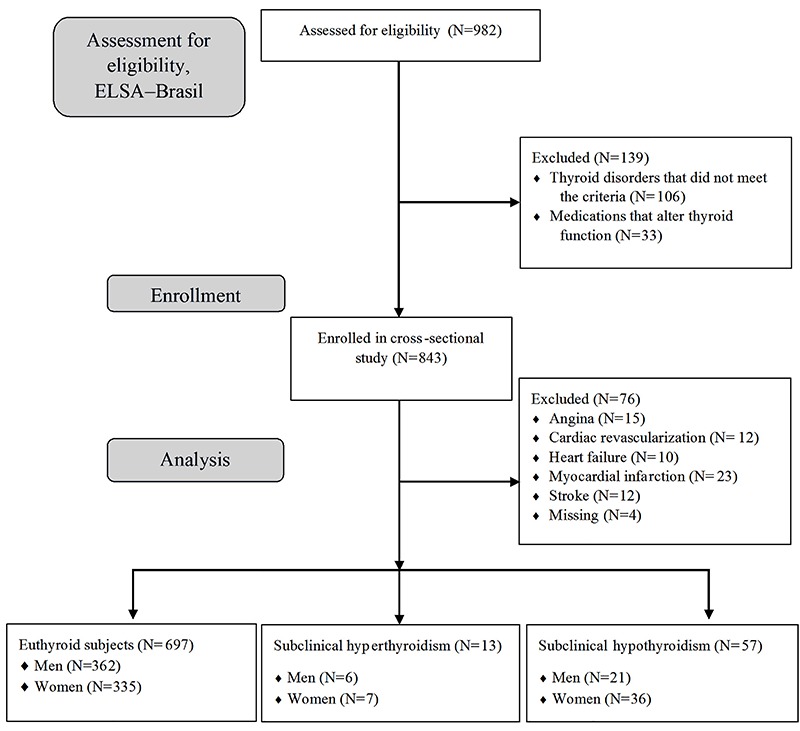
Study flow-chart. CCTA: coronary computed tomography angiography. Medications (n=33): propranolol (n=23), primidone (n=2), carbamazepine (n=2), lithium (n=2), amiodarone (n=1), furosemide (n=1), levodopa (n=1), and valproic acid (n=1); phenytoin, carbidopa, metoclopramide, heparin, haloperidol, interferon (n=0).

We included 982 asymptomatic subjects with no previous history of cardiovascular disease who underwent a CCTA. Details about exclusions are presented in the Figure 1. After exclusions, we included 767 subjects, classified as euthyroid (n=697; 90.9%), SCHypo (n=57; 7.4%) or SCHyper (n=13; 1.7%). Median age was 58 (IQR=55-63) and 378 participants (49.3%) were women. Median radiation exposure was 3.60 mSv (IQR=3.19-4.75).

Table 1 shows characteristics of groups according to quintiles of TSH. Quintile cutoffs were as follows: 0.00-0.95 mIU/L (1st), 0.96-1.32 mIU/L (2nd), 1.33-1.78 mIU/L (3rd), 1.79-2.71 mIU/L (4th), and 2.72-35.5 mIU/L (5th). Among subjects with SCHypo, median TSH was 5.06 mIU/L (IQR=4.36-6.28 mIU/L), among subjects with SCHyper, 0.19 mIU/L (IQR=0.05-0.33 mIU/L), and among euthyroid subjects was 1.50 mIU/L (IQR=1.03-2.21 mIU/L), P<0.0001. Only three subjects had TSH above 10 mIU/L.

**Table 1.** General characteristics of the sample according to the quintiles of thyrotropin (TSH).

Quintiles of TSH	1st (n=155)	2nd (n=151)	3rd (n=154)	4th (n=154)	5th (n=153)	P for trend
	0.00–0.95	0.96–1.32	1.33–1.78	1.79–2.71	2.72–35.5	
Age (years)^+^	58 (56–63)	59 (55–62)	58 (55–63)	58 (55–63)	58 (55–63)	0.455
Women (n, %)	82 (52.9)	62 (41.1)	79 (51.3)	67 (43.5)	88 (57.5)	0.372
Race (n, %)						<0.001
White	81 (54.0)	79 (53.0)	89 (58.2)	102 (67.1)	100 (68.0)	
Mixed	26 (17.3)	22 (14.8)	35 (22.9)	29 (19.1)	24 (16.3)	
Black	28 (18.7)	30 (20.1)	17 (11.1)	9 (5.9)	16 (10.9)	
Other**	15 (10.0)	18 (12.1)	12 (7.8)	12 (7.9)	7 (4.8)	
Body mass index* (kg/m^2^)	27.2 (4.7)	27.3 (4.6)	27.8 (4.7)	26.7 (4.2)	28.0 (5.1)	0.380
Hypertension (n, %)	76 (49.0)	60 (39.7)	60 (39.0)	68 (44.2)	56 (36.6)	0.103
Diabetes mellitus (n, %)	43 (27.7)	45 (29.8)	41 (26.6)	42 (27.3)	37 (24.2)	0.398
Dyslipidemia (n, %)	100 (64.9)	92 (61.3)	98 (64.1)	94 (61.8)	91 (59.5)	0.399
HDL-cholesterol (mg/dL)*	58.0 (14.2)	57.3 (15.5)	56.9 (13.9)	56.5 (16.2)	56.9 (15.4)	0.427
Triglycerides (mg/dL)^+^	114 (85–154)	119 (81–167)	122 (85–167)	126 (90–170)	125 (97–183)	0.004
Smoking (n, %)						0.014
Never	68 (43.9)	70 (46.4)	74 (48.1)	80 (51.9)	75 (49.0)	
Past	49 (31.6)	59 (39.1)	61 (39.6)	48 (31.2)	65 (42.5)	
Current	38 (24.5)	22 (14.6)	19 (12.3)	26 (16.9)	13 (8.5)	
GFR (mL·min^-1^/1.73m^2^)*	83.8 (15.8)	80.4 (14.3)	82.9 (15.2)	81.0 (14.2)	80.2 (14.5)	0.089
C-reactive protein (mg/L)^+^	1.5 (0.7–3.0)	1.5 (0.7–3.3)	1.5 (0.8–3.6)	1.1 (0.7–2.4)	1.5 (0.7–3.1)	0.754
10-year CHD risk (n, %)^+^	8 (4–13)	8 (5–13)	7 (4–13)	8 (5–11)	8 (4–13)	0.991
Coronary artery disease (n, %)	89 (57.4)	80 (53.0)	77 (50.0)	92 (59.7)	76 (49.7)	0.491
SIS >4 (n, %)	24 (15.5)	21 (13.9)	22 (14.3)	26 (16.9)	15 (9.8)	0.346
SSS >4 (n, %)	27 (17.4)	24 (15.9)	22 (14.3)	29 (18.8)	18 (11.8)	0.367

CHD: coronary heart disease risk by Framingham Risk Score; GFR: glomerular filtration rate by CKD-Epi; SIS: segment involvement score; SSS: segment severity score. ^+^Median and interquartile range; *mean±SD; **Asian and Indigenous. Statistical analysis was done with ANOVA.

Individuals in the highest TSH quintiles reported a higher proportion of White race and of non-smokers. Subjects in the highest TSH quintiles tended to have higher values of triglycerides. Frequency of CAD, SIS>4, and SSS>4 were similar among the quintiles of TSH.

Table 2 shows adjusted odds ratio (OR) for presence of CAD. In full-adjusted logistic regression models, neither of the extreme quintiles were associated with CAD (adjusted OR=1.29, 95%CI=0.79-2.13, and OR=0.95, 95%CI=0.58-1.56 for the 1st and 5th quintile compared to the 3rd, model 2). Restricting analysis to euthyroid participants did not change the results (adjusted OR=1.43; 95%CI=0.84-2.43, and OR=1.15, 95%CI=0.69-1.93). Tables 3 and 4 show the results for the association with SIS>4 and SSS>4, respectively, according to TSH quintiles. No association was found among 1st and 5th quintiles of TSH and SIS>4 (OR=0.94, 95%CI=0.46-1.90, and OR=0.82, 95%CI=0.38-1.73) nor SSS>4 (OR=1.17, 95%CI=0.59-2.33 and OR=0.98, 95%CI=0.48-2.02). Restricting analysis to euthyroid subjects did not significantly alter these results.

**Table 2.** Odds ratio and 95% confidence interval for associations of coronary artery disease and quintiles of thyrotropin (TSH).

Quintiles of TSH	Crude	Model 1	Model 2
Euthyroid, SCHypo, SCHyper (n=767)			
Coronary artery disease			
1st (0.00–0.95 mIU/L)	1.35 (0.86–2.11)	1.43 (0.89–2.31)	1.29 (0.79–2.13)
2nd (0.96–1.32 mIU/L)	1.13 (0.72–1.77)	1.05 (0.65–1.69)	0.98 (0.59–1.60)
3rd (1.33–1.78 mIU/L)	1.0 (reference)	1.0 (reference)	1.0 (reference)
4th (1.79–2.71 mIU/L)	1.48 (0.95–2.33)	1.38 (0.86–2.22)	1.38 (0.84–2.27)
5th (2.72–35.5 mIU/L)	0.99 (0.63–1.54)	0.96 (0.60–1.54)	0.95 (0.58–1.56)
P (1st *vs* 3rd)	0.191	0.139	0.313
Euthyroid subjects only (n=697)			
Coronary artery disease			
1st (0.41–0.94 mIU/L)	1.46 (0.91–2.35)	1.57 (0.95–2.60)	1.43 (0.84–2.43)
2nd (0.95–1.28 mIU/L)	1.22 (0.76–1.95)	1.14 (0.69–1.88)	1.02 (0.61–1.71)
3rd (1.29–1.67 mIU/L)	1.0 (reference)	1.0 (reference)	1.0 (reference)
4th (1.68–2.44 mIU/L)	1.59 (0.99–2.55)	1.56 (0.95–2.57)	1.49 (0.89–2.52)
5th (2.45–3.97 mIU/L)	1.24 (0.78–1.99)	1.18 (0.72–1.95)	1.15 (0.69–1.93)
P (1st *vs* 3rd)	0.117	0.079	0.192

SCHypo: subclinical hypothyroidism; SCHyper: subclinical hyperthyroidism. Model 1: adjusted for age, gender, race/skin color; Model 2: model 1 plus hypertension, diabetes mellitus, dyslipidemia, smoking, BMI, HDL-cholesterol, triglycerides, glomerular filtration rate by CKD-Epi (Chronic Kidney Disease Epidemiology Collaboration), and C-reactive protein.

**Table 3.** Odds ratio and 95% confidence interval for associations of segment involvement score (SIS) >4 and quintiles of thyrotropin (TSH) levels.

Quintiles of TSH	Crude	Model 1	Model 2
Euthyroid, SCHypo, SCHyper (n=767)			
SIS >4			
1st (0.00–0.95 mIU/L)	1.10 (0.59–2.06)	1.16 (0.60–2.27)	0.94 (0.46–1.90)
2nd (0.96–1.32 mIU/L)	0.97 (0.51–1.85)	0.87 (0.44–1.71)	0.75 (0.37–1.53)
3rd (1.33–1.78 mIU/L)	1.0 (reference)	1.0 (reference)	1.0 (reference)
4th (1.79–2.71 mIU/L)	1.22 (0.66–2.26)	1.07 (0.56–2.06)	1.14 (0.58–2.24)
5th (2.72–35.5 mIU/L)	0.65 (0.32–1.31)	0.71 (0.34–1.48)	0.82 (0.38–1.73)
P (1st *vs* 3rd)	0.767	0.659	0.855
Euthyroid subjects only (n=697)			
SIS >4			
1st (0.41–0.94 mIU/L)	0.91 (0.47–1.75)	0.94 (0.47–1.91)	0.68 (0.32–1.45)
2nd (0.95–1.28 mIU/L)	0.89 (0.46–1.72)	0.77 (0.38–1.54)	0.60 (0.29–1.25)
3rd (1.29–1.67 mIU/L)	1.0 (reference)	1.0 (reference)	1.0 (reference)
4th (1.68–2.44 mIU/L)	1.05 (0.56–2.00)	0.96 (0.49–1.87)	0.90 (0.45–1.82)
5th (2.45–3.97 mIU/L)	0.60 (0.29–1.23)	0.61 (0.29–1.28)	0.61 (0.28–1.33)
P (1st *vs* 3rd)	0.776	0.872	0.316

SCHypo: subclinical hypothyroidism; SCHyper: subclinical hyperthyroidism. Model 1: adjusted for age, gender, race/skin color; Model 2: model 1 plus hypertension, diabetes mellitus, dyslipidemia, smoking, BMI, HDL-cholesterol, triglycerides, glomerular filtration rate by CKD-Epi (Chronic Kidney Disease Epidemiology Collaboration), and C-reactive protein.

**Table 4.** Odds ratio and 95% confidence interval of association of SSS >4 and quintiles of TSH levels.

Quintiles of TSH	Crude	Model 1	Model 2
Euthyroid, SCHypo, SCHyper (n=767)			
SSS >4			
1st (0.00–0.95 mIU/L)	1.27 (0.69–2.34)	1.37 (0.71–2.63)	1.17 (0.59–2.33)
2nd (0.96–1.32 mIU/L)	1.13 (0.61–2.12)	1.03 (0.53–2.00)	0.94 (0.47–1.86)
3rd (1.33–1.78 mIU/L)	1.0 (reference)	1.0 (reference)	1.0 (reference)
4th (1.79–2.71 mIU/L)	1.39 (0.76–2.55)	1.23 (0.65–2.33)	1.31 (0.68–2.53)
5th (2.72–35.5 mIU/L)	0.80 (0.41–1.56)	0.89 (0.44–1.79)	0.98 (0.48–2.02)
P (1st *vs* 3rd)	0.451	0.346	0.651
Euthyroid subjects (n=697)			
SSS >4			
1st (0.41–0.94 mIU/L)	1.07 (0.57–2.03)	1.15 (0.58–2.29)	0.91 (0.44–1.88)
2nd (0.95–1.28 mIU/L)	1.05 (0.56–2.00)	0.93 (0.47–1.82)	0.78 (0.38–1.57)
3rd (1.29–1.67 mIU/L)	1.0 (reference)	1.0 (reference)	1.0 (reference)
4th (1.68–2.44 mIU/L)	1.17 (0.62–2.19)	1.06 (0.55–2.07)	1.03 (0.52–2.04)
5th (2.45–3.97 mIU/L)	0.75 (0.38–1.48)	0.77 (0.38–1.58)	0.78 (0.37–1.63)
P (1st *vs* 3rd)	0.829	0.683	0.789

SCHypo: subclinical hypothyroidism; SCHyper: subclinical hyperthyroidism. Model 1: adjusted for age, gender, race/skin color; Model 2: model 1 plus hypertension, diabetes mellitus, dyslipidemia, smoking, BMI, HDL-cholesterol, triglycerides, glomerular filtration rate by CKD-Epi (Chronic Kidney Disease Epidemiology Collaboration), and C-reactive protein.

We performed an analysis with FT4 as independent variables. Results are available in Supplementary Table S1 with the distribution of general characteristics by FT4 quintiles. Table 5 shows adjusted OR for the presence of CAD, SIS>4, and SSS>4. No association was found among 1st and 5th quintiles of FT4 for three independent variables: (OR=1.16, 95%CI=0.70-1.90, and OR=0.90, 95%CI=0.55-1.47), (OR=1.39, 95%CI=0.66-2.94, and OR=0.88, 95%CI=0.42-1.87), and (OR=1.19, 95%CI=0.58-2.46, and OR=0.81, 95%CI=0.39-1.68), respectively.

**Table 5.** Odds ratio and 95% confidence interval of association of coronary artery disease and quintiles of free thyroxine (FT4).

Quintiles of FT4	Crude	Model 1	Model 2
Coronary artery disease (all=746)*			
1st (0.8–1.00 ng/dL)	1.12 (0.72–1.75)	1.10 (0.68–1.78)	1.16 (0.70–1.90)
2nd (1.01–1.08 ng/dL)	1.04 (0.66–1.62)	1.06 (0.66–1.70)	1.03 (0.63–1.68)
3rd (1.09–1.16 ng/dL)	1.0 (reference)	1.0 (reference)	1.0 (reference)
4th (1.17–1.25 ng/dL)	1.09 (0.69–1.72)	0.98 (0.60–1.59)	0.95 (0.57–1.58)
5th (1.26–2.11 ng/dL)	1.12 (0.72–1.76)	1.02 (0.63–1.63)	0.90 (0.55–1.47)
P (1st *vs* 3rd)	0.626	0.687	0.571
SIS>4 (n=746)			
1st (0.8–1.00 ng/dL)	1.13 (0.58–2.20)	1.23 (0.60–2.52)	1.39 (0.66–2.94)
2nd (1.01–1.08 ng/dL)	0.96 (0.48–1.93)	1.04 (0.50–2.16)	0.97 (0.45–2.10)
3rd (1.09–1.16 ng/dL)	1.0 (reference)	1.0 (reference)	1.0 (reference)
4th (1.17–1.25 ng/dL)	1.76 (0.93–3.33)	1.70 (0.87–3.33)	1.88 (0.93–3.78)
5th (1.26–2.11 ng/dL)	1.08 (0.55–2.12)	0.92 (0.45–1.88)	0.88 (0.42–1.87)
P (1st *vs* 3rd)	0.731	0.567	0.382
SSS>4 (n=746)			
1st (0.8–1.00 ng/dL)	1.00 (0.52–1.94)	1.07 (0.53–2.16)	1.19 (0.58–2.46)
2nd (1.01–1.08 ng/dL)	1.16 (0.61–2.21)	1.28 (0.65–2.53)	1.24 (0.61–2.52)
3rd (1.09–1.16 ng/dL)	1.0 (reference)	1.0 (reference)	1.0 (reference)
4th (1.17–1.25 ng/dL)	1.95 (1.06–3.57)	1.92 (1.01–3.64)	2.07 (1.07–4.03)
5th (1.26–2.11 ng/dL)	0.96 (0.49–1.87)	0.82 (0.40–1.65)	0.81 (0.39–1.68)
P (1st *vs* 3rd)	0.992	0.842	0.638

* All includes euthyroid, subclinical hypothyroidism and subclinical hyperthyroidism. Model 1: adjusted for age, gender, race/skin color; Model 2: model 1 plus hypertension, diabetes mellitus, dyslipidemia, smoking, BMI, HDL-cholesterol, triglycerides, glomerular filtration rate by CKD-Epi (Chronic Kidney Disease Epidemiology Collaboration), and C-reactive protein. SIS: segment involvement score; SSS: segment severity score.

## Discussion

Our study found no association between TSH and FT4 levels and presence, extent, or severity of coronary artery disease. Findings did not change after restricting analysis to euthyroid participants.

Contrasting with our results, Park et al. (7), in the largest study to date evaluating the association of CAD using CCTA with euthyroid and SCHypo subjects, found a strong and independent association between presence of CAD and SCHypo (adjusted OR=2.13; 95%CI=1.05-4.03). This was a retrospective analysis in South Korea that included 2404 asymptomatic outpatients mostly men and 49 (2%) participants with SCHypo. Similar to our study, they used CCTA for CAD diagnosis, which was defined as a presence of any degree of plaque detected in at least one segment. Different from our analysis, those authors studied in a larger sample the association of CAD with SCHypo and we used TSH quintiles. Furthermore, they included a higher frequency of men (97 *vs* 50.3% in our analysis), higher 10-year CHD risk scores (mean 15 *vs* 10.0% in our study) and subjects with SCHypo who had higher TSH levels compared to the present analysis (mean of 7.52 *vs* 6.22 mIU/L in our study). That study has no data about FT4 levels and CAD.

Rodondi et al. (3), in a meta-analysis of 55,287 participants with normal thyroid function or with SCHypo from 11 prospective cohorts, showed an independent association between SCHypo and CHD events only in a sensitive analysis in subjects with TSH levels higher than 10 mIU/L. Differences between our results for higher levels of TSH compared to the meta-analysis of that study might be partially explained by the small number of subjects with SCHypo in our sample with TSH values higher than 10 mUI/l (n=2).

We have also found that lower levels of TSH were not associated with CAD. Our data contrasts with findings from a large meta-analysis by Collet et al. (2), which evaluated CHD events among subjects with SCHyper in comparison with euthyroid ones. They included 52,674 participants from 10 cohort studies with available data about SCHyper, incident CHD, and mortality. However, none of these cohorts detected occult CAD using CCTA (2). SCHyper was independently associated with CHD events (HR=1.21; 95%CI=0.99-1.46), all-cause mortality (HR=1.29; 95%CI=1.02-1.62), and cardiovascular mortality (HR=1.24, 95%CI=1.06-1.46) (2). Another prospective study with a mean follow-up around 3 years, 76 middle-aged subjects with SCHyper and 1062 euthyroid ones, all of them with type 2 diabetes mellitus, showed a higher incidence of CHD events with lower TSH levels. Subjects with TSH <0.1 mIU/L presented HR=4.96 (95%CI=1.01-25.66, P for trend=0.049) for CHD events in comparison with subjects in the reference category, or with TSH between 0.45 and 4.49 mIU/L, regardless of glycemic control (22). In our sample, only 0.5% (n=4) of the total number of subjects were SCHyper with TSH values lower than 0.1 mUI/L.

We did not find that TSH levels were associated with extent or severity of CAD in an asymptomatic population. Auer et al. used cineangiocoronariography to evaluate the relationship of subclinical thyroid disorders and coronary heart disease in 100 consecutive subjects, 90 euthyroid, 6 with SCHyper, and 4 with SCHypo (59% men and mean of age 63.7 years) (8). Contrasting with our results, they found an independent association between higher levels of TSH and severity of coronary arteries involvement, defined as two or three-vessel obstructive disease (8). However, due to the inclusion of symptomatic patients, their sample had a high frequency (35%) of double or triple-vessel disease (8). Meanwhile, our analysis included only asymptomatic subjects and only 15.6% of subjects had SSS>4, a proxy for multiple vessel disease.

Evidence from the literature shows an association of subclinical thyroid diseases and subclinical atherosclerosis (9,10). Two large South Korean cross-sectional studies have demonstrated an association between increased values of CAC among euthyroid subjects and low-normal levels of TSH (9) and FT4 (9,10), suggesting a U-shaped curve between thyroid function and CAC. However, other cross-sectional studies have found negative results between CAC and SCHypo (23,24), but have detected associations only in high-risk subgroups, such as liver steatosis (23) and men aged 55 years with a Framingham Heart Score ≥10% (24). Previous analysis of ELSA-Brasil reported an association of low and low-normal TSH levels with CAC>100 among euthyroid subjects and individuals with subclinical thyroid disorders (25). In women, but not in men, we observed a U-shaped relationship between CAC>100 and TSH quintiles (25). We also found a weak association between intima media thickness and SCHypo (α=0.010; 95%CI=0.0004-0.019; P=0.041) (26). Differences among these studies and the present analysis may be explained by the smaller sample that underwent CCTA compared to CAC and intima media thickness and the relatively young mean age of the sample with a positive association with subclinical atherosclerosis but not with clinical obstruction of coronary arteries.

This study has limitations. A cross-sectional design does not allow evaluating causality. Therefore, our results must be considered within the context of the study design. TSH was measured only once and we did not have FT3 levels available for all samples. Our study also had some strengths. The ELSA-Brasil is a multicenter, large cohort study that used strict protocols and validated questionnaires in the baseline assessment. Although this study has a negative result, this is the third study to date that evaluated the relationship between TSH levels and CAD, detected by CCTA. We have included participants from a mostly healthy adult population, with similar proportion of males and females, and information about several cardiovascular risk factors and other covariates.

In conclusion, in a sample of healthy middle-aged adults without overt thyroid disease, we did not find an independent association between the TSH and FT4 levels and occult CAD. Furthermore, TSH and FT4 levels were also not found to be independently associated with severity of coronary artery obstruction or number of segments involved.

## Supplementary Material

Click here to view [pdf]

## Conflicts of interests

E.J.F. Peixoto de Miranda is a medical manager at Bayer Pharmaceuticals AG, Brazil. The other authors report no conflicts of interest.
